# Cardiovascular Action of Insulin in Health and Disease: Endothelial L-Arginine Transport and Cardiac Voltage-Dependent Potassium Channels

**DOI:** 10.3389/fphys.2016.00074

**Published:** 2016-03-15

**Authors:** Sebastián Dubó, David Gallegos, Lissette Cabrera, Luis Sobrevia, Leandro Zúñiga, Marcelo González

**Affiliations:** ^1^Department of Kinesiology, Faculty of Medicine, Universidad de ConcepciónConcepción, Chile; ^2^Vascular Physiology Laboratory, Department of Physiology, Faculty of Biological Sciences, Universidad de ConcepciónConcepción, Chile; ^3^Department of Morphophysiology, Faculty of Medicine, Universidad Diego PortalesSantiago, Chile; ^4^Cellular and Molecular Physiology Laboratory (CMPL), Division of Obstetrics and Gynecology, Faculty of Medicine, School of Medicine, Pontificia Universidad Católica de ChileSantiago, Chile; ^5^Department of Physiology, Faculty of Pharmacy, Universidad de SevillaSeville, Spain; ^6^Faculty of Medicine and Biomedical Sciences, University of Queensland Centre for Clinical Research (UQCCR), University of QueenslandHerston, QLD, Queensland, Australia; ^7^Centro de Investigaciones Médicas, Escuela de Medicina, Universidad de TalcaTalca, Chile; ^8^Group of Research and Innovation in Vascular Health (GRIVAS-Health)Chillán, Chile

**Keywords:** insulin, L-arginine, nitric oxide, endothelium, cardiac potassium channels, ventricular repolarization, heart failure, insulin resistance

## Abstract

Impairment of insulin signaling on diabetes mellitus has been related to cardiovascular dysfunction, heart failure, and sudden death. In human endothelium, cationic amino acid transporter 1 (hCAT-1) is related to the synthesis of nitric oxide (NO) and insulin has a vascular effect in endothelial cells through a signaling pathway that involves increases in hCAT-1 expression and L-arginine transport. This mechanism is disrupted in diabetes, a phenomenon potentiated by excessive accumulation of reactive oxygen species (ROS), which contribute to lower availability of NO and endothelial dysfunction. On the other hand, electrical remodeling in cardiomyocytes is considered a key factor in heart failure progression associated to diabetes mellitus. This generates a challenge to understand the specific role of insulin and the pathways involved in cardiac function. Studies on isolated mammalian cardiomyocytes have shown prolongated action potential in ventricular repolarization phase that produces a long QT interval, which is well explained by attenuation in the repolarizing potassium currents in cardiac ventricles. Impaired insulin signaling causes specific changes in these currents, such a decrease amplitude of the transient outward K^+^ (Ito) and the ultra-rapid delayed rectifier (IKur) currents where, together, a reduction of mRNA and protein expression levels of α-subunits (Ito, fast; Kv 4.2 and IKs; Kv 1.5) or β-subunits (KChIP2 and MiRP) of K^+^ channels involved in these currents in a MAPK mediated pathway process have been described. These results support the hypothesis that lack of insulin signaling can produce an abnormal repolarization in cardiomyocytes. Furthermore, the arrhythmogenic potential due to reduced Ito current can contribute to an increase in the incidence of sudden death in heart failure. This review aims to show, based on pathophysiological models, the regulatory function that would have insulin in vascular system and in cardiac electrophysiology.

## General overview of cardiovascular regulation by insulin in health and disease

Insulin is a peptide hormone produced by the β cells, of the islets of Langerhans. Functionally, is the most potent anabolic hormone, promoting the synthesis and storage of carbohydrates, lipids and proteins, while inhibiting their degradation and release into circulation. This hormone stimulates the uptake of glucose, amino acids, and fatty acids; and increases the expression of enzymes involved in the glycogen, lipid, and protein synthesis. At the same time, it reduces the activity of the enzymes that catalyse degradation of same molecules (Saltiel and Kahn, 2001). Cellular effects of insulin are mediated by the activation of insulin receptors (IRs A/B), and a signaling pathway involved in the regulation of nutrients metabolism, mitochondrial biogenesis, cellular grown, proliferation, differentiation, and migration (Nystrom and Quon, 1999). IRs are glycoproteins consisting of an extracellular α-subunit (135 kDa) and a transmembrane β-subunit (95 kDa). These receptors are allosteric enzymes in which the α-subunit regulates tyrosine kinase activity of the β-subunits. The binding of insulin to the α-subunit results in dimerization, forming the α_2_β_2_ complex in the plasma membrane. The complex α_2_β_2_ generates the autophosphorylation of β-subunit at tyrosine (Tyr) 1158 (Tyr^1158^), Tyr^1162^, and Tyr^1163^, the first step in the activation cascade induced by insulin (Patti and Kahn, 1998). Phosphorylation of IR induces the tyrosine kinase activity toward insulin receptor substrate (IRS) 1 (IRS-1) or 2 (IRS-2), generating binding sites for Src homology 2 (SH2) domain proteins, including phosphatidylinositol 3-kinase (PI3K), RAS guanine nucleotide exchange factor complex known as growth factor receptor-bound protein 2/son of sevenless (GRB2/SOS), and SH2 domain-containing protein tyrosine phosphatase-2 (SHP2) and other SH2 proteins (Copps and White, 2012). These proteins provide specific docking sites for the recruitment of downstream signaling proteins, leading to activation of mitogen-activated protein kinase (MAPK), protein kinase B (PKB/Akt) and protein kinase C (PKC) signaling cascades (White, 2003; Schultze et al., 2012).

Since the early nineties, the cardiovascular actions of insulin have been studied in humans, and the main information was collected in several works published by Alain Baron and Helmut Steinberg. In 1990, in a study made in obese and lean subjects determined a correlation between insulin resistance (hyperglycaemia and hyperinsulinemia), and lack of vasodilatory effects of insulin in leg blood flow (LBF). These obese subjects showed a lower capacity for uptake D-glucose and changes in cardiac function, such as reduction in cardiac index [cardiac output (CO)/body surface area] and stroke volume (SV) without change in heart rate (HR) (Baron et al., 1990). Authors concluded that insulin resistance could be a result of insulin's actions both in glucose uptake and hemodynamic regulation, highlighting the *in vivo* role of insulin as an endocrine regulator of cardiovascular physiology. Later, healthy subjects under a protocol of euglycemic hyperinsulinemia showed increases of CO, SV, HR, and LBF; and decreases of mean arterial pressure (MAP), systemic vascular resistance (SVR), and leg vascular resistance (LVR). Correlation analysis showed that the insulin-mediated glucose uptake is inversely proportional to MAP or SVR, supporting the hemodynamic basis for insulin resistance (Baron et al., 1993). The effects of insulin are part of physiological regulation of cardiovascular system, with a delicate interaction with other signals, like norepinephrine (NE) and acetylcholine (Ach). Obese insulin-resistant subjects show more sensitivity to contractile effects of NE, and lower clearance of NE in response to insulin (Baron et al., 1994). More recently, a reduction was shown in Ach-dependent forearm blood flow in obese patients, but this effect disappears in the group of obese subjects without metabolic syndrome (Schinzari et al., 2015). Even though it is not possible to discard a deleterious effect of obesity *per se* on the cardiovascular system, mechanisms that we discuss in this review show that insulin resistance could be a primary cause of cardiovascular disease in metabolic disorders, leading to a deterioration of endothelium-dependent vasodilatation and higher sensibility to vasoconstrictors, like NE or other locally released molecules. The accumulated evidence shown by the literature makes it possible to establish that insulin-dependent blood flow increments are blunted in obese, and type 2 diabetes mellitus (T2DM) patients, conditions associated with insulin resistance. Physiological regulation of blood flow by insulin depends on a mechanism that involves the activation of PI3K, PKB, and nitric oxide (NO) synthesis (Baron, 2002; Steinberg and Baron, 2002). Due to the increasing prevalence of diabetes and insulin resistance, a main objective of this article is to make a critical review of physiological mechanisms regulated by insulin both in vasculature and in the heart. Thus, we can propose a hypothesis for pathological mechanisms of cardiovascular disease in diabetes or insulin resistance.

Diabetes mellitus (DM) is characterized by chronic hyperglycaemia which results from defects in insulin secretion (type 1 DM), insulin action (T2DM), or both (WHO, 1998). Diabetes is a common disease that affects ~382 million people worldwide, and the costs to society are high and escalating. By the end of 2013, diabetes caused 5.1 million deaths and cost USD 548 billion in healthcare spending (IFD, 2013). Although treatment has been improved over the last decades, diabetes is related with several complications like acute hyperglycaemic or hypoglycaemic events, kidney disease, eye disease, peripheral vascular disease, coronary artery disease, cerebrovascular disease, and congestive heart failure (Bethel et al., 2007).

Cardiovascular diseases (CVD) represent the leading causes of morbidity and mortality in patients with diabetes (Gregg et al., 2014), with cardiac and vascular dysfunctions as key steps of the pathophysiological mechanism. Vascular dysfunction is primarily caused by endothelial dysfunction, a phenomenon related with deterioration in the capacity of these cells to synthesize NO. NO is synthesized from the semi-essential cationic amino acid L-arginine and molecular oxygen (O_2_) by endothelial NO synthase (eNOS), leading to the formation of NO and the neutral amino acid L-citrulline (Palmer et al., 1988). The enzyme depends on several cofactors, including Ca^2+^/calmodulin complex, tetrahydrobiopterin (BH_4_), nicotinamide adenine dinucleotide phosphate (NADPH), flavin adenine dinucleotide (FAD), and flavin mononucleotide (FMN) (Knowles and Moncada, 1994; Sessa, 1994). In absence of NO signaling there is a disturbance in vascular homeostasis, triggering a series of events leading to pathologies like as hypertension, hypercholesterolemia, renal vascular insufficiency, and chronic heart failure (Vanhoutte, 2003; Wierzbicki et al., 2004; Yang et al., 2007). As mentioned above, several studies in diabetic patients have demonstrated deficiency in endothelium-dependent vasodilation, a disturbance that generates an imbalance in vascular tone, which ultimately leads to the development of endothelial dysfunction (Williams et al., 1996; Rask-Madsen and King, 2007). Mechanisms underlying the decrease in NO-dependent dilatation in diabetes include decreased bioavailability of tetrahydrobiopterin (BH4) cofactor and uncoupling of eNOS (Cai et al., 2005), increased activity of arginase (an enzyme that competes with the eNOS for L-arginine; Vanhoutte and Tang, 2008), high levels of asymmetric dimethylarginine (ADMA, endogenous inhibitor of eNOS; Xiong et al., 2003); increased production of superoxide anion (${\text{O}}_{2}^{-}$) and peroxynitrite (ONOO^−^) synthesis (Cosentino et al., 2003; Quijano et al., 2007), inactivation of NO by advanced glycation end products (Gao et al., 2008) and abnormal responses of vascular smooth muscle cells (VSMC) (Lesniewski et al., 2008; Shi and Vanhoutte, 2008).

On the other hand, a main cardiac disorder is diabetic cardiomyopathy (DCM), a pathology associated with alterations in molecular metabolism and structure of cardiomyocytes (Fang et al., 2004). These changes include, among others, a ventricular electric remodeling characterized by long-term alterations in ion channel activity that could be a compensatory or adaptive mechanism (Casis and Echevarria, 2004). Electrical remodeling has been related with contractile failure due to abnormal increase of intracellular Ca^2+^ concentration or to arrhythmias that lead to sudden death (Straus et al., 2006). Most electrical disturbances are related to the abnormal repolarization of cardiac action potential (Shimoni et al., 2004). Electrophysiological studies have clearly identified the different types of voltage-dependent currents (inward or outward currents), which contribute to the repolarization phase in the mammalian myocardium (Nerbonne and Kass, 2005). Outward currents are clearly differentiated and cardiac myocyte express voltage-dependent K^+^ channels (Kv) that contribute to the development of action potential (Nerbonne, 2000). Changes in the functional properties of the Kv channels can generate dramatic effects in myocardial action potential, and also in the normal cardiac rhythm generation (Lengyel et al., 2007). Cardiovascular complications in diabetes have been associated to a series of electrocardiographic disturbances, such as QT interval prolongation, even in short-term diabetes (Veglio et al., 2002a,b; Zhang et al., 2011). Experimental evidence suggests that specific remodeling of some K^+^ channels in the ventricle is related with these alterations (Casis and Echevarria, 2004).

## Insulin regulation of vascular system

### Relevance of L-arginine transport in vascular physiology

In endothelial cells, uptake of the substrate L-arginine for NO synthesis is mediated by transport systems called y^+^, y^+^L, b^0, +^, and B^0, +^. The main transport system involved in this process is the system y^+^, responsible for ~85% of L-arginine transport in physiological state (Devés and Boyd, 1998). This system includes the family of proteins known as cationic amino acids transporters (CATs), formed by CAT-1, CAT-2A, CAT-2B, CAT-3, and CAT-4 (Verrey et al., 2004; Casanello et al., 2007). Members of the CATs family are encoded by genes *SLC7A* 1, 2, 3, and 4 (Verrey et al., 2004). CAT-1, encoded by *SLC7A1* gene, is ubiquitously expressed, while CAT-2A and CAT-3 are constitutively expressed in liver and brain, respectively. CAT-2B is induced under inflammatory conditions in a variety of cells including T cells and macrophages. The CAT-4 gene sequence is 41–42% identical to the other members of the family CATs, but its transport activity has not been described. The function of CATs was suggested because of their structural similarity to *Saccharomyces cerevisiae*, permease for L-histidine and L-arginine. Its function was verified by expression in *Xenopus laevis oocytes* for kinetic transport studies (Closs et al., 1997). CAT-1, CAT-2B, and CAT-3 are Na^+^ independent transporters, presenting medium affinity for its substrate (*K*_m_ ~50–250 μM), whereas CAT-2A has low affinity for cationic amino acids (*K*_m_ ~2–5 mM) (Devés and Boyd, 1998; Palacín et al., 1998; Mann et al., 2003).

In cytoplasm, L-arginine is used for synthesis of proteins, NO, urea, creatine, agmatine, polyamines, and other molecules. Intracellular concentration of L-arginine is near to 1 mM, but the *K*_m_ of eNOS by L-arginine is ~3 μM and, as mentioned, the *K*_*m*_ of hCAT-1/2B for L-arginine is ~50–250 μM (Shin et al., 2011). These inconsistencies between cytoplasmic concentrations of L-arginine and the affinity for substrate of L-arginine/NO system, constitutes the “L-arginine paradox.” This paradox is under discussion among researchers, especially given the growing relevance of L-arginine transport from extracellular space and the concept of compartmentalization of different pools of L-arginine (Karbach et al., 2011; Simon et al., 2013). In a simple but very elegant experiment, Shin et al. demonstrated that L-arginine transport mediated by a facilitated diffusion system is absolutely necessary for NO synthesis: when EA.hy926 endothelial cells are incubated with modified L-arginine, which has the ability to enter cells by passive diffusion, eNOS is not able to synthesize NO. Additionally, in knockdown cells for hCAT-1 (induced by siRNA), L-citrulline synthesis is significantly reduced, showing the relevance of this specific transporter (Shin et al., 2011). This evidence is supported by hCAT-1 localization studies. In porcine aortic endothelial cells (PAEC) CAT-1 colocalize with eNOS and caveolin-1 (Cav-1, structural protein of caveolae; McDonald et al., 1997). The subcellular localization through overexpression of CAT-1 fused to green fluorescent protein (GFP) has been studied in different cell lines (Closs et al., 2006). In hamster kidney baby cells (HKB) it has been noted that CAT-1-GFP is localized predominantly in caveolin associated domains in plasma membrane (Lu and Silver, 2000). In another study, CAT-1-EGFP was mainly found in intracellular vesicles of U373MG, human glioblastoma cells, and to a lesser extent a lesser extent in the plasma membrane (Wolf et al., 2002). In Madin-Darby canine kidney epithelial cells (MDCK) and human embryonic kidney 293 cells (HEK293), the expression of CAT-1-GFP was detected, predominantly in the basolateral plasma membrane (Cariappa et al., 2002; Kizhatil and Albritton, 2002). Meanwhile, more research into HEK293 showed that mCAT-1-GFP is located in filopodia, and near to Golgi (Masuda et al., 1999). Recently, Guo et al. showed that CAT-1-GFP it expressed in plasma membrane in colocalization with VE-caherin and the authors propose that CAT-1 have a role as cellular adhesion molecule (CAM) and that the incubation with extracellular L-arginine augments L-arginine transport via promoting the CAT-1 shift from endothelial cells junctions to the free surface of ECs (Guo et al., 2015). Therefore, the L-arginine paradox has a potential explanation in the characteristics of CAT-1 cell surface expression, generating functional clusters for ensure an adequate supply of L-arginine for eNOS activity. Due to their physiological relevance, we focus on determining changes induced by insulin and/or diabetes on activity and expression of CAT-1.

### Regulation of hCAT-1 by insulin in normoglycaemic or hyperglycaemic environment

Insulin increases the mRNA expression levels of hCAT-1 and hCAT-2B in human umbilical vein endothelial cells (HUVEC), correlates with high rate of L-arginine transport and NO synthesis (González et al., 2004) in a mechanism that involves high abundance of the protein in the plasma membrane (González et al., 2011). Insulin exerts a vasodilatory effect (González et al., 2011; Guzmán-Gutiérrez et al., 2012) through a signaling pathway involving PI3K, PKB/Akt, and MAPK activity that activates hCAT-1 and eNOS in endothelial cells (González et al., 2004). It has been described that this stimulation by insulin is caused by the activation of the promoter of the *SLC7A1*, gene encoding hCAT-1, via a mechanism that involves multiple binding sequences for the transcriptional factor specificity protein 1 (Sp1), located between 117 and 105 upstream base pairs from the transcription start site (TSS) (González et al., 2011). The *SLC7A1* promoter belongs to the TATA-less group, so binding sites for Sp1 located near the TSS would be responsible for both basal expression and regulation of gene expression to stimulation by growth factors (Sobrevia and González, 2009). These effects have been observed with physiologic concentrations of insulin (0.1–10 nM), so we propose that the expression of *SLC7A1* and hCAT-1 activity would be under a tonic regulation by physiological levels of plasma insulin. Moreover, it is reported that similar concentrations of insulin (1 μM) induces an acute (5 min) increase of L-arginine transport by a mechanism dependent on PI3K activity and PKC in human aortic endothelial cells (HAEC) (Kohlhaas et al., 2011)

**Figure 1:**
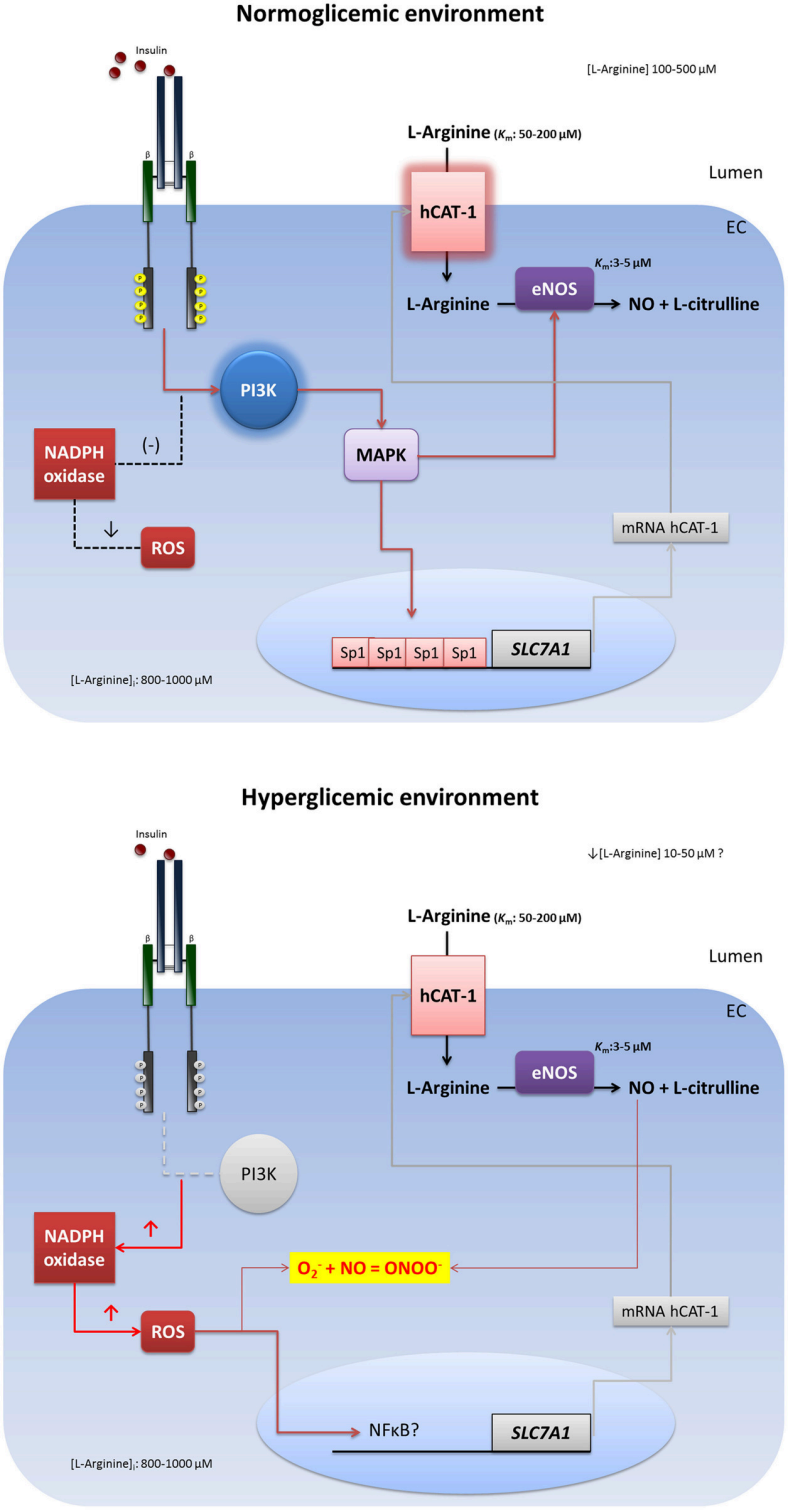
**Impairment insulin signaling in endothelial hyperglycemic environment**. In physiological state, insulin induces a signaling pathway that involves the activation of phosphatidylinositol 3-kinase (PI3K), mitogen activated protein kinase (MAPK), and specificity protein 1 (Sp1). This pathway induces the transcriptional activity of the *SLC7A1* gene and the plasma membrane expression of hCAT-1 for increased the L-arginine transport and nitric oxide (NO) synthesis in endothelial cells. In hyperglycaemia, insulin resistance causes deterioration of intracellular signaling that reduces the transcriptional activity of *SLC7A1*, diminishing the L-arginine transport and NO synthesis. Also the insulin resistance abolishes the antioxidant capacity of the hormone, increasing the reactive oxygen species (ROS) and lowering the bioavailability of NO through the reaction with superoxide (${\text{O}}_{2}^{-}$) to form peroxynitrite (ONOO^−^).

As was mentioned above, diabetes is related with hyperglycaemia and insulin resistance, which are conditions that stimulate the oxidative stress in vasculature. Furthermore, exposure of HUVEC to high extracellular concentrations of D-glucose increases the synthesis of ${\text{O}}_{2}^{-}$ dependent of NAD(P)H oxidase, which reacts with the NO to generate ONOO^−^, contributing to endothelial dysfunction (Sobrevia and González, 2009; González et al., 2015). Long-term incubation (24 h) of HUVEC with 25 mM D-glucose increases the transport of L-arginine and cGMP accumulation in a similar magnitude to that observed in HUVEC from pregnancies with gestational diabetes mellitus (Sobrevia et al., 1996, 1997). Increased transport of L-arginine in both chronic incubation with D-glucose and gestational diabetes has been linked to increased mRNA levels for hCAT-1 and eNOS activity (Vásquez et al., 2004, 2007). In HAEC, prolonged incubation (7 days) with 25 mM D-glucose induces decreased eNOS activity (determined by nitrite content) and lower abundance of the protein and mRNA level (Furfine et al., 1994). This effect is associated with decreased activity of eNOS promoter (Srinivasan et al., 2004). In HUVEC, the hyperglycaemic environment induces an increase in the abundance of the eNOS protein (Vásquez et al., 2007), an effect that would be associated with the activity of a signaling pathway involving the PI3K and Akt. It has also been shown that in BAEC, there is a reduced production of insulin-dependent NO when cells were incubated with high extracellular concentration of D-glucose, an effect that seems to depend on a signaling pathway involving the type 1 insulin receptor (IR-1), PI3K and the inhibitor of nuclear factor kappa-B kinase subunit beta (IKKβ) (Kim et al., 2006). Furthermore, increased production of cGMP induced by D-glucose in HUVEC is blocked by incubating the cells with 1 nM insulin (Sobrevia et al., 1997); and the incubation with 1 nM insulin (8 h) is sufficient to block the effect of D-glucose on the decrease of adenosine transport (Muñoz et al., 2006), an important vasoactive nucleoside (San Martín and Sobrevia, 2006). More recently, González et al. (2015) demonstrated that high extracellular concentration of D-glucose increases the expression of hCAT-1 and L-arginine transport, inducing high synthesis of NO associated with NADPH oxidase-dependent ${\text{O}}_{2}^{-}$ synthesis in HUVEC. On other hand, insulin (in normoglycaemic environment) increases L-arginine transport and NO synthesis without changes in ${\text{O}}_{2}^{-}$ levels, showing a physiological response that results in relaxation of umbilical vein (González et al., 2011). Interestingly, the effect of high D-glucose is associated with high contractile response to U46619 (thromboxane A2 analog) and hydrogen peroxide (H_2_O_2_). Co-incubation with insulin recovers the adequate response to vasoconstrictors and decreases the NADPH oxidase-dependent ROS (González et al., 2015). These mechanisms induced by high D-glucose could be responsible for endothelial dysfunction in diabetes, a scenario in which the L-arginine and NO convert from “good guys” to “bad guys” in company with high levels of ROS. In these conditions, insulin reduces the oxidative stress to improve bioavailability of NO, as long as the endothelial cell retains its capacity to respond appropriately to the hormone (Figure 1). In fact, in diabetic patients there is a significant reduction of forearm blood flow in response to insulin infusion, and an increase in L-arginine clearance (L-arginine incorporation to the tissue) during insulin infusion was 41% less in diabetic subjects compared to controls after adjusting for covariates age, BMI, MAP, and HDL cholesterol (Rajapakse et al., 2013). The data discussed in this section confirm the potential association between T2DM (and gestational diabetes), insulin resistance and endothelial dysfunction. Hence, further research must be focused on detailed mechanisms that regulate the cardiovascular actions of insulin in other states, such as hypertension and heart failure. Following this principle, in the second part of this article we review the effects of disturbances in cardiac insulin signaling.

## Insulin regulation of cardiac function

### Overview about action potential of cardiomyocytes

**Figure 2:**
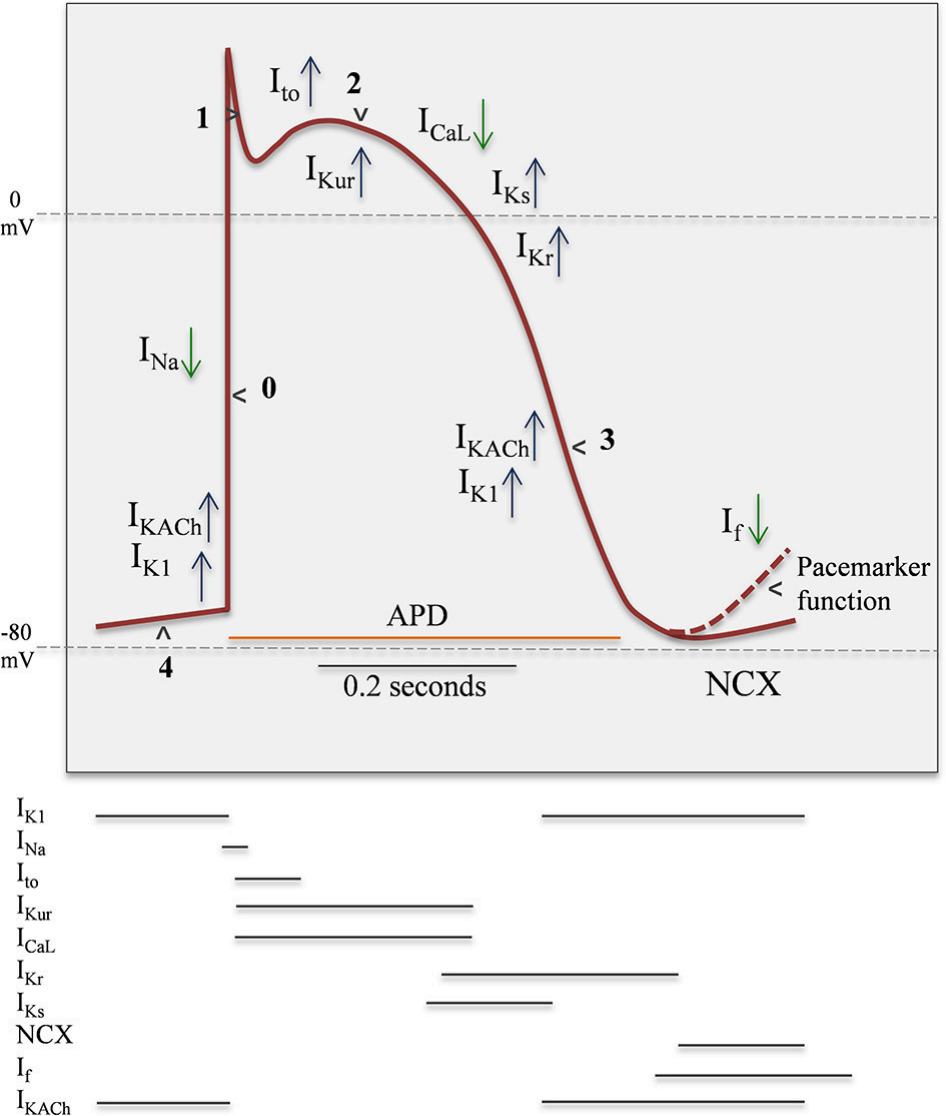
**Representation of ventricular Action Potential (AP) and the temporal ionic current contribution**. Blue arrow up represents outward currents, green arrow down represents inward currents. AP have five well-differenced states: 4, Resting; 0, Upstroke; 1, Early repolarization; 2, Plateau; 3, Final repolarization. The time- and voltage-dependent properties of the voltage-gated inward Na^+^ (Nav) and Ca^2+^ (Cav) currents expressed in human atrial and ventricular myocytes are similar. In contrast, there are multiple types of K^+^ currents, particularly Kv currents, contributing to ventricular action potential repolarization. The properties of the various Kv currents are distinct, and in contrast to the inward currents, there are multiple Kv currents expressed in individual myocytes throughout the myocardium. IK1, inward rectifier current; Ito, transient outward current; IK, delayed rectification currents: Ikur, ultra-rapid; Ikr, rapid; Iks, slow; ICaL, L-type Ca^2+^ current; Ina, sodium current; NCX, Na^+^/Ca^2+^ exchanger; If, funny current; IKACh, G-protein-activated K^+^ current.

Normal mechanical function of the heart in mammals depends on proper electrical functioning, which is reflected in the sequential activation of pacemaker cells and subsequent electrical propagation through the ventricles. Coordinated electrical functioning of the heart is recorded by a surface electrocardiogram (ECG). Propagation activity and electromechanical coordination of ventricles also depends on coupling among cells, mediated by gap junctions (Kanno and Saffitz, 2001). Generation of action potential (AP) in myocardium results from sequential activation and inactivation of ion channels, which can lead input currents (Na^+^ and Ca^2+^) and an output repolarization currents (K^+^) (Figure 2) (Antzelevitch and Dumaine, 2002; Nerbonne and Kass, 2005).

AP differ in distinct regions of the heart, and contribute to the spreading of excitation way through the myocardium and the subsequent generation of normal heart beat (Kléber and Rudy, 2004). Changes in functional properties, or in the expression of ion channels in the myocardium, as a result of mutations (Antzelevitch, 2003) or myocardial diseases (Cesario et al., 2006), can lead to changes in the AP, synchronization or spread, including the heart's predisposition to potentially life-threatening arrhythmias (Akar et al., 2000; Casis et al., 2000; Casis and Echevarria, 2004). Given the clinical relevance of heart rhythm, it is of considerable interest to analyse the cellular and molecular mechanisms that contribute to the generation and maintenance of normal heart rhythm. Animal non-human studies suggests that small changes in time- and voltage-dependence properties of ion channels, in the myocardial sarcolemma, may have profound effects in both the action potentials time as well in the refractory period and rhythmicity (Delmar, 1992; Delisle et al., 2004).

In atrial myocytes, ventricular myocytes and Purkinje fibers, the onset of AP is fast (Phase 0) and given principally by voltage-dependent Na^+^ (Nav) channels activity (Fozzard, 2002). In contrast, in pacemaker cells of sino-atrial (SA) node (SAN) and atrio-ventricular (AV) node (AVN), this phase 0 is markedly slower. Phase 0 in Purkinje fibers and myocytes is followed by a transient repolarization (phase 1), reflecting Nav channel inactivation and the activation of the fast transient voltage-gated outward K^+^ current (Ito, f) (Niwa and Nerbonne, 2010). This transient repolarization, which can be quite prominent in Purkinje and ventricular cells, influences the shape and the time of the action potential plateau (phase 2). Plasma membrane depolarization also activates voltage-gated Ca^2+^ (Cav) currents, and the subsequent influx of Ca^2+^ through L-type Cav channels, during the phase 2 (*plateau)*, triggering the excitation-contraction coupling in myocardium (Bers and Perez-Reyes, 1999). The driving force for K^+^ efflux is high during the *plateau* phase, and when Cav channels are inactivated, the outward K^+^ currents predominate, resulting in repolarization (phase 3). As a result, the voltage of plasma membrane returns to the resting potential. In contrast to Nav and Cav currents, there are multiple types of voltage-gated K^+^ (Kv) currents, as well as non-voltage-gated, inwardly rectifying K^+^ (Kir) currents (Figure 2 and Table 1). At least two types of transient outward currents, Itof and Itos, and several delayed rectifiers including IKr (IK rapid), IKs (IK slow), and IKur (IK ultra-rapid) have been distinguished (Brunet et al., 2004). The time- and voltage-dependent properties of Kv currents identified in myocytes from different species and/or from different regions of the heart present a remarkable similarity, suggesting that the same (or very similar) molecular entities contribute to the generation of each of the various types of Kv channels (Table 1) in different cells/species.

**Table 1:**
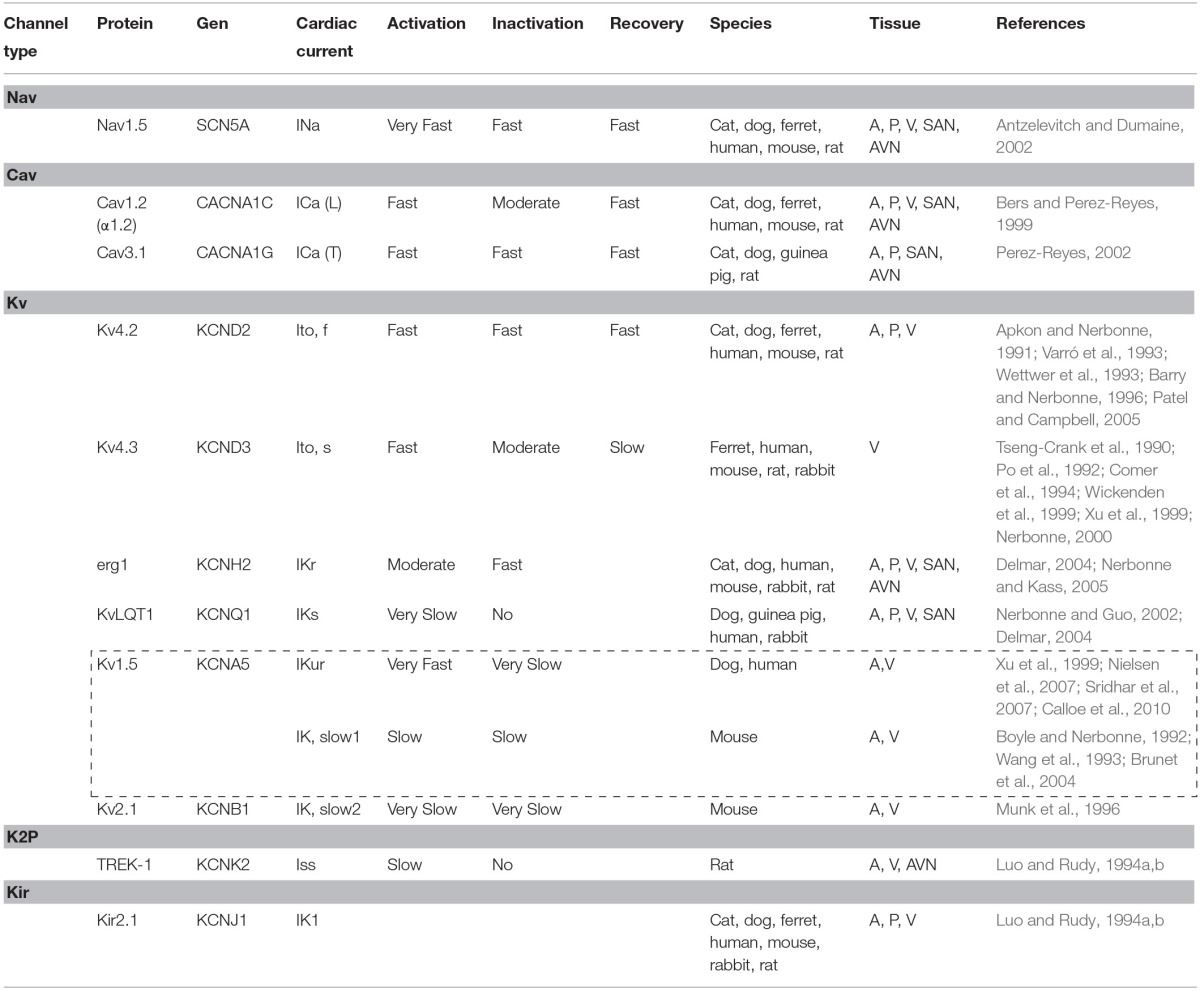
**Cardiac currents contributing to action potential repolarization**.

*A, atrial; P, Purkinje; V, ventricular; SAN, sinoatrial node; AVN, atrioventricular node. The dashed boxes are placed around current given different names in different species that likely are encoded by the same channel's subunit genes*.

### Voltage-dependent potassium channels

Based on biophysical differences and pharmacological sensitivities there are two main classes of Kv currents: transient outward currents (Ito) and delayed rectifier (IK) currents (Barry and Nerbonne, 1996; Nerbonne and Guo, 2002). The Ito currents are activated and rapidly inactivated on a depolarization stimuli (~−30 mV) and underlie the early phase (phase 1) of repolarization in ventricular and atrial cells in rat (Apkon and Nerbonne, 1991), mouse (Benndorf and Nilius, 1998), cat (Furukawa et al., 1990), rabbit (Hiraoka and Kawano, 1989), dog (Litovsky and Antzelevitch, 1998), and humans (Varró et al., 1993; Wettwer et al., 1993). The Ito currents influence Cav channel activation and the balance between inward and outward currents during phase 2. Cardiac IK currents are activated at similar plasma membrane potentials and present variable kinetics. These currents are determinant for the latter phase of repolarization that determine the diastolic potential (Barry and Nerbonne, 1996).

The two components of the Ito currents identified in the heart cell types show different kinetics of recovery (Xu et al., 1999). The Ito rapid current component (Ito fast, Itof) is recovered rapidly in the range of 60–100 ms (Patel and Campbell, 2005). In contrast, the slow component (Ito slow, Itos) recovers slowly with time constants in the order of seconds (Xu et al., 1999). Both are differentially expressed and contribute to heterogeneity of the action potential shape (Brunet et al., 2004).

**Figure 3:**
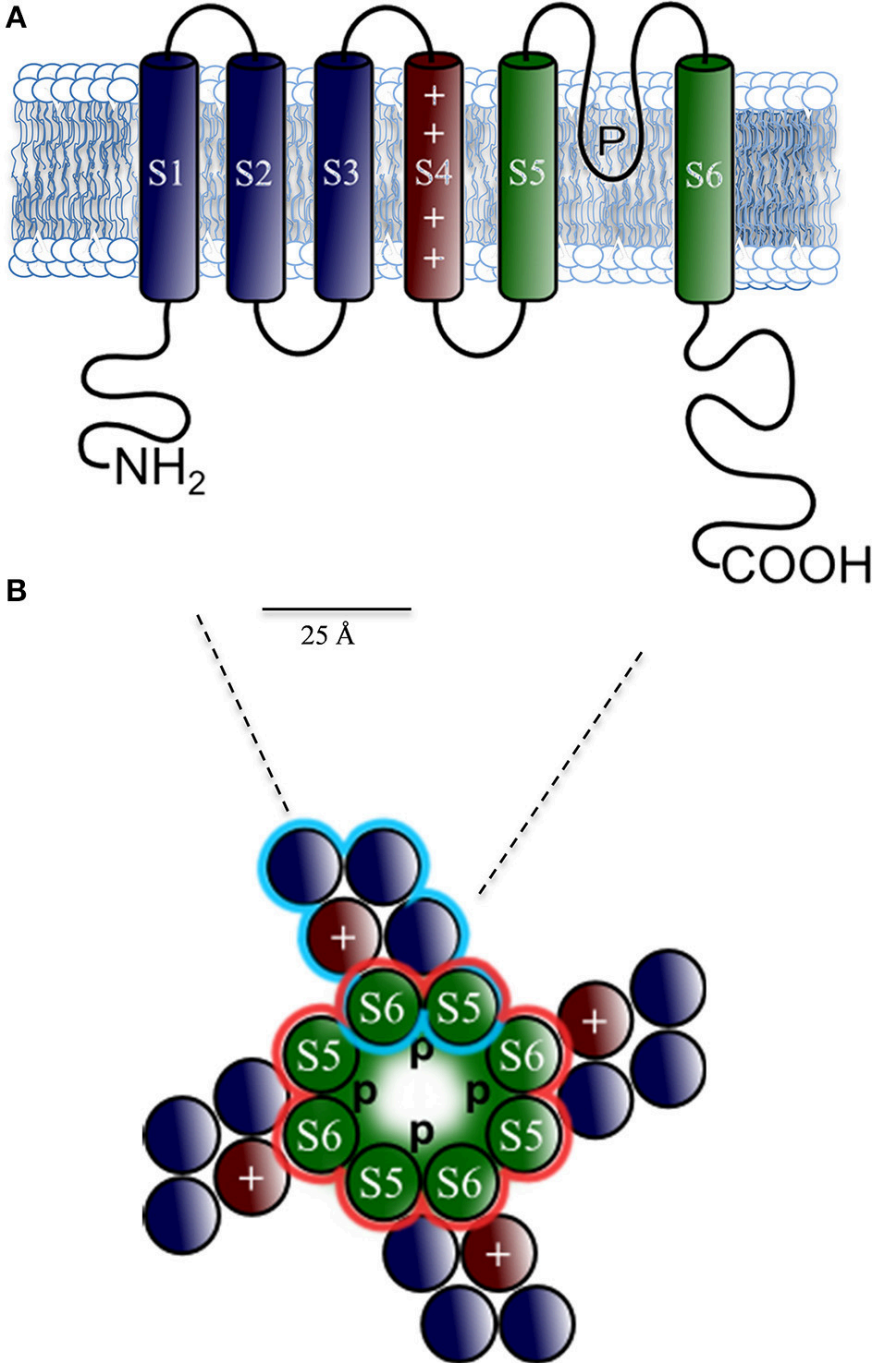
**Predicted protein topology of cardiac K^+^−channel α subunits**. Schematic representations of **(A)** the six-transmembrane (S1–S6) one P-loop (6Tm-1P) voltage-dependent K^+^−channel (Kv) α-subunits topology that conduct IKur, Ito, Iks, IKr, and If in the heart; in red voltage sensor (S4); in green the pore region formed by S5 and S6 segments. Note the amino (NH2) and carboxyl (COOH) terminal tails located intracellular, which serves as assembly sites for β-subunits as KCHiP2. **(B)** Structure of the tetrameric assembly of the Kv Channel viewed from the extracellular side. The S5-P-loop-S6 region assembles with three other pore domains (central P) functions as a K^+^ selectivity filter.

Structurally, Kv channels are comprised of four α subunits that form the channel pore (MacKinnon, 1991), each subunit with six transmembrane segments (denoted S1–S6) including the voltage sensor S4 (Snyders, 1999), a single pore region between S5 and S6, and C- and N-terminal intracellular domains. The pore region works as a K^+^ selectivity filter (Doyle et al., 1998). In Kv1 to Kv4, the amino acids sequence at the N-terminal domain, which precedes S1, plays a critical role in the assembly Kvα subunit (Figure 3) (McKeown et al., 2008). Evidence suggests that Kv α-subunits of the Kv4 subfamily are responsible for a rapidly activating, inactivating, and recovering cardiac transient outward Kv channels referred to as Itof (Table 1). In rat and mouse ventricular myocytes exposed to antisense oligodeoxynucleotides targeted against Kv4.2 or Kv4.3, Itof density is reduced by < 50% (Fiset et al., 1997; Guo et al., 2002). Significant reductions in rat ventricular Itof density are also seen in cells exposed to an adenoviral construct encoding a truncated Kv4.2 subunit (Kv4.2ST) that functions as a negative dominant (Johns et al., 1997). In addition, it has been reported that Itof is eliminated in ventricular myocytes isolated from transgenic mice, expressing a pore mutant of Kv4.2, W362F (Barry et al., 1998). Taken together, these results demonstrate that members of the Kv4 subfamily underlie Itof in mouse and rat ventricles. Biochemical studies have also shown that Kv4.2 and Kv4.3 are associated in adult mouse ventricles, suggesting that functional ventricular Itof reflect the heteromeric assembly of the Kv4.2 and Kv4.3 α-subunits (Guo et al., 2002). In dog and human myocardium, however, Kv4.2 appears not to be expressed (Kong et al., 1998), suggesting that only Kv4.3 contributes to Itof, in larger mammals. Moreover, the genetic deletion of Kv4.2 abolishes Itof current, revealing a critical role of Kv4.2 channels in generating these currents in rodents (Guo et al., 2005).

Biophysical properties of slow transient outward myocardial Kv currents, referred as Itos, are different. Considerable evidence supports that myocardial Itos currents are encoded by the Kv1 α-subunit, Kv1.4. Heterologous expression of Kv1.4 yields slowly recovering (seconds), 4-aminopyridine (4-AP, Kv inhibitor) sensitive transient Kv currents (Tseng-Crank et al., 1990; Po et al., 1992; Comer et al., 1994; Wickenden et al., 1999) that closely resemble Itos (Fermini et al., 1992; Brahmajothi et al., 1999; Wang et al., 1999; Xu et al., 1999). In addition, expression of Kv1.4 has been demonstrated at the transcript level in rat (Tseng-Crank et al., 1990; Roberds and Tamkun, 1991; Wickenden et al., 1999), ferret (Brahmajothi et al., 1999), canine (Dixon et al., 1996), and human (Tamkun et al., 1991) hearts, and at the protein level in mouse, rat and ferret ventricles (London et al., 1998; Brahmajothi et al., 1999; Wickenden et al., 1999) and rabbit atrium (Wang et al., 1999). Direct support for this hypothesis was complemented on myocytes isolated from (Kv1.4^−∕−^) mice with a targeted disruption in the *kcna4* (Kv1.4) locus (London et al., 1998). Waveforms of outward currents in cells isolated from Kv1.4^−∕−^ ventricles are indistinguishable from those recorded in wild-type ventricular cells (Guo et al., 1999). In cells isolated from the interventricular septum from Kv1.4^−∕−^, however, Itos is undetectable, thereby demonstrating that Kv1.4 underlies Itos (Guo et al., 1999).

Some studies suggest that additional subunits are required for the generation of cardiac Ito currents. Indeed, a number of molecularly diverse accessory subunits can modulate biophysical properties and cell surface expression of heterologously-expressed Kv4 channels (Guo et al., 2000; Abbott et al., 2007). Of these accessory subunits, only the cytoplasmic KChIP2 protein has been shown directly to be an essential component of myocardial Itof (Grubb et al., 2012). Co-expression of KChIP subunits dramatically increases the cell surface expression of Kv4 channels, Itos inactivation and markedly accelerates recovery (An et al., 2000). Also, KChIP2 co-immunoprecipitates with Kv 4.2 (and Kv4.3) α subunits in adult mouse ventricles (Guo et al., 2002) and the targeted deletion of the KChIP2 locus (*kcnip2*) abrogates ventricular Itof (Kuo et al., 2001). Interestingly, KChIP2 protein expression is dramatically reduced in Kv4.2^−∕−^ ventricles (Guo et al., 2005), suggesting that expression of the Kv4 and KChIP2 proteins are reciprocally regulated. Other regulatory subunit that colocalize with Kv4.2 is the MinK-related peptide (MiRP) (McCrossan and Abbott, 2004; Roepke et al., 2008). Although MiRP does not affect the densities of expressed Kv4.2-KChIP2 currents (Liu et al., 2008), targeted deletion of the gene encoding MiRP result in reduced (< 25%) ventricular Itof densities without changes in total or surface Kv4.2 expression (Roepke et al., 2008).

The ultra-rapid potassium current IKur is well expressed in the atria, where it contributes to repolarization (Amos et al., 1996). The molecular constituent of IKur is the Kv1.5 potassium channel (Boyle and Nerbonne, 1992; Wang et al., 1993). Direct experimental support for this hypothesis was provided with the demonstration that exposure to antisense oligonucleotide targeted against Kv1.5 selectively attenuates IKur in isolated adult human (Snyders et al., 1993; Feng et al., 1997) and rat atrial myocytes (Bou-Abboud and Nerbonne, 1999). The important physiological role for Kv1.5 in human atria is suggested by the finding that IKur densities and Kv1.5 protein expression are reduced markedly in atria of patients with chronic atrial fibrillation (Van Wagoner et al., 1997). Although, IKur has predominantly been reported in atria, it has also been suggested to play a role in canine and human ventricles (Nielsen et al., 2007; Sridhar et al., 2007; Calloe et al., 2010).

### Insulin regulation of cardiac electrical function

Insulin has a direct effect in cardiomyocytes, in glucose transport and oxidation, glycolysis, glycogen and protein synthesis (Abel, 2004; Bertrand et al., 2008). *In vivo*, many of insulin's effects on cardiac function and metabolism are related to systemic responses such as an increment in peripheral and coronary vasodilatation, and sodium and water intake by the kidney (Muniyappa et al., 2007).

Insulin is a key regulator of cardiac growth as reported in studies carried out in cardiac insulin receptor knockout (CIRKO) mice. In this study, the cardiac index was reduced by 22–28%, possibly due to a decrease in myocyte size rather than a reduction in cell number (Belke et al., 2002). The role of insulin in cell size regulation is supported by studies in *Drosophila* with disrupted homolog of vertebrate insulin signaling, were the knock-out model for IRS, had less than half the size of wild-type flies, owing to fewer and smaller cells (Böhni et al., 1999). In experimental mice with targeted deletion of IRS-1 or IRS-2, there has been evidence of a reduction in postnatal cardiac size (Araki et al., 1994), which is indicative of conserved roles for insulin signaling in the determination of cardiac growth. Inhibition of PI3K function in mouse heart using a negative dominant transgene, results in a similar phenotype generated in CIRKO mice (Shioi et al., 2000). Negative dominant PI3K transgenic mice showed 16% reduction in cardiac index and myocyte surface area reduction of 18% (Belke et al., 2002). Thus, impaired PI3K activity represents a mechanism that explains the cardiac size phenotype in CIRKO mice.

In addition, insulin could increase cardiac contractility, affecting cardiac output, and mediates the cellular hypertrophy and generates an antiapoptotic effect on cardiomyocytes by activating other intermediary intracellular signaling pathways (Belke et al., 2002). Changes in these pathways and in the pathologic processes linked to status of insulin deficiency or insulin resistance could contribute to an increased risk of cardiac hypertrophy, pathological remodeling of the left ventricle and heart failure, among others (Cesario et al., 2006).

Diverse evidences suggests that the stimulatory effect of insulin involves K^+^ channel protein synthesis, which is responsible for maintaining both Ito and IKur currents; although it is proposed that the hormone regulates differentially these two currents (Shimoni et al., 1998; Rozanski and Xu, 2002; Lengyel et al., 2007; Torres-Jacome et al., 2013). In a recent review, Ballou et al. collected and summarized several studies that shows that PI3K signaling regulates many aspects of proteins involved in AP in the heart, including protein expression, trafficking and gating Nav1.5, Kv11.1, Kv7.1, and Cav1.2 in cardiomyocytes. This evidence comes from studies where drugs that inhibit PI3K signaling cause QT prolongation, but the role of PI3K in transcriptional regulation of genes in cardiomyocytes is relatively unexplored (Ballou et al., 2015).

However, a study of isolated cardiomyocytes from T1DM mice induced by STZ and treated with wortmannin (inhibitor of PI3K) prior to incubation with insulin, showed that insulin is still able to restore the densities of Ito currents without effect on other potassium currents (Ui et al., 1995). This indicates that this hormone could not induce its effects through PI3K pathway in T1DM (Shimoni et al., 1998). Insulin stimulates another important signaling pathway, MAPK/ERK, which can lead to cell proliferation and protein synthesis (Guo, 2014); furthermore, its activation can be blocked by PD98059, a specific inhibitor of the phosphorylation of ERK1/2 (Lazar et al., 1995). In isolated cardiomyocytes from T1DM rats treated with PD98059 (30 min) prior to the addition of insulin, blockage is perceived in the insulin-dependent return of the densities of potassium Ito currents (Shimoni et al., 1998). These results suggest that the effects on the maintenance of these currents and their respective channels occur in a MAP-kinase dependent process (Shimoni et al., 1998).

### Cardiac disease in diabetes mellitus

Studies have found that diabetic patients have an increased risk of CVD (Gregg et al., 2014). Common complications are acute myocardial infarction (181.5 events/1000 people/year) and stroke (126.2 events/1000 people/year; Bethel et al., 2007). In a cohort of geriatric American adults with T2DM between 2004 and 2010, cardiovascular complications have the highest incidence, followed by eye disease and hypoglycaemic events (Huang et al., 2014). The presence of diabetes is associated with a high risk of cardiac failure (Aneja et al., 2008), and is present in the 75% of patients with idiopathic dilated cardiomyopathy (Bertoni et al., 2003). The “Framingham Heart” Study (Kannel and McGee, 1979) showed that the incidence of heart failure is 2 and 5 times higher for diabetic men and women, respectively. Diabetic patients with macrovascular complications showed a strong association with cardiomyopathy, and its association is well correlated with the duration and severity of hyperglycaemia (Poornima et al., 2006).

#### Diabetic cardiomyopathy

DM affects cardiac structure and function, which under certain conditions can lead to a condition called DCM. Rubler et al. described this condition first in 1972 and defined it as “ventricular dysfunction that occurs independently of coronary artery disease and hypertension” (Rubler et al., 1972). In addition, DCM is characterized by diastolic dysfunction, which becomes more apparent in presence of hypertension or myocardial ischemia (Fang et al., 2004). In humans, the DCM is also characterized by diastolic dysfunctions, which precede the development of systolic dysfunction (Liu et al., 2001). These findings also could be found in patients with T2DM, where diastolic dysfunction affected 30% of the patients (Boudina and Abel, 2007).

The effects of DCM lead to cardiomegaly, left ventricular dysfunction, ventricular electrical remodeling and symptoms of congestive heart failure (Casis et al., 2000). The resulting electrophysiological changes contribute to an increase in the incidence of cardiac arrhythmias and sudden death (Casis et al., 2000). Electrical changes have been associated with the surface ECG abnormalities, including T and QT wave prolongation (Casis et al., 2000; Casis and Echevarria, 2004).

Insulin resistance in the heart is a factor that contributes significantly to DCM (Mandavia et al., 2013). Therefore, it is important to distinguish between the secondary effects to environmental disturbances in states of insulin resistance and specific changes that occur in the signaling pathways that are intrinsic to heart tissue. These effects determine, among others, changes in cardiomyocyte AP as might be the extension of repolarization phase (Lengyel et al., 2007), and thus infer the pathophysiological mechanism that would play insulin on heart function.

### Morphological changes in the ventricular action potential

**Figure 4:**
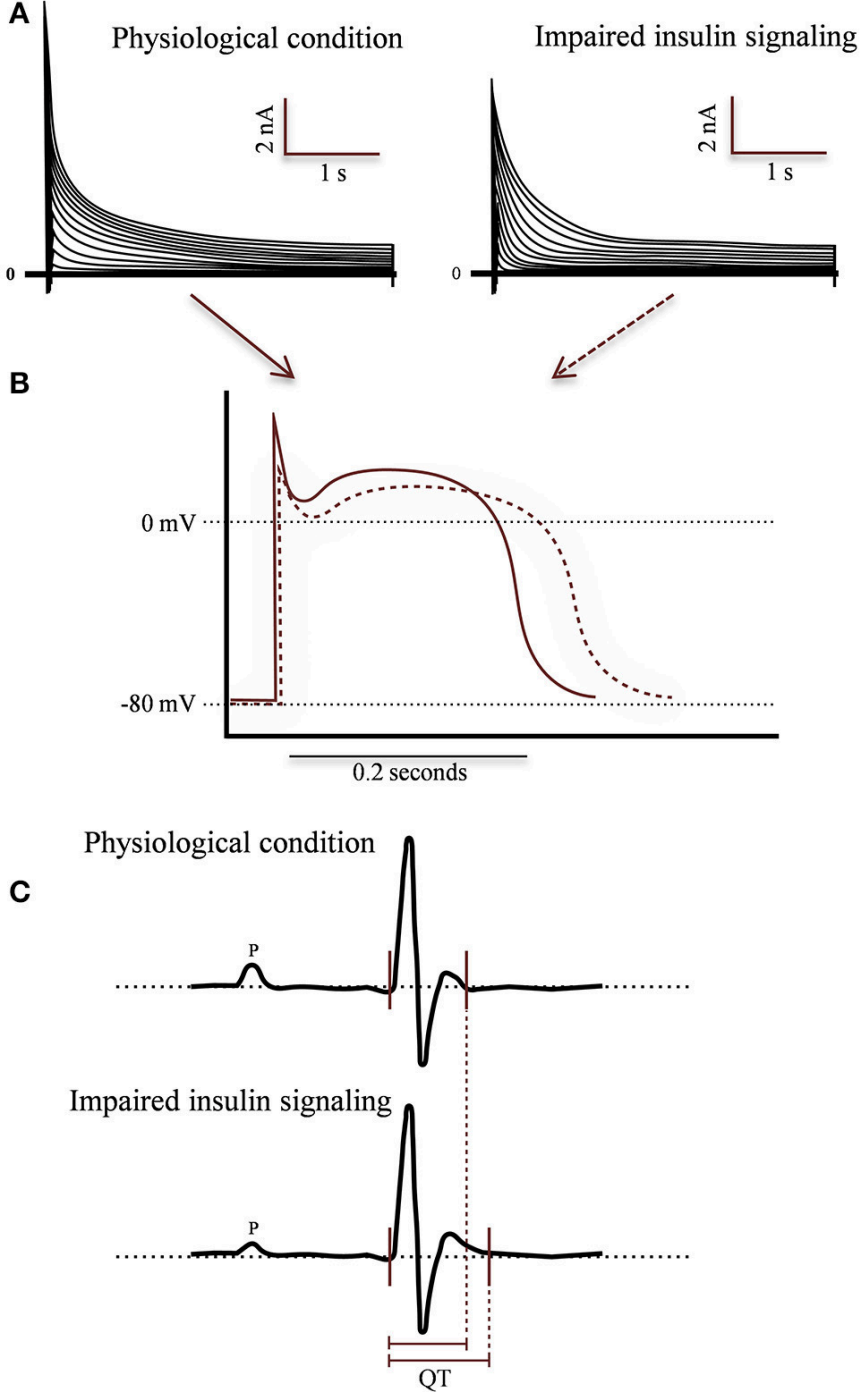
**Morphological changes associated to impaired insulin in different experimental models: T1DM, T2DM, and CIRKO**. **(A)** Representative whole cell voltage-gated outward K currents recorded from Wild-type (WT) animal (left) and impaired insulin signaling condition (right). Outward K^+^ currents are reduced in ventricle of experimental models. **(B)** Representative action potentials waveforms recorded from left ventricular myocytes isolated from WT (solid red line) and experimental mice (dashed red line). Insulin signaling alterations produce a prolonged action potential duration (APD) in experimental ventricular cells. Effect of absent or impaired cardiac insulin signaling at the level of the whole organism by measuring surface ECG activity in anesthetized mice. In **(C)** is illustrate the comparison of ECG recordings from WT and experimental mice. Analyses of the ECG parameters confirmed a significant prolongation mainly in QT intervals in experimental relative to control littermates. Relative differences in QT intervals are indicated below the records by red solid lines.

The morphology of AP is clearly altered in T1DM and T2DM, showing a prolonged QT in myocytes isolated from STZ models in either, mice (Magyar et al., 1992; Casis et al., 2000) or rabbits (Torres-Jacome et al., 2013). AP duration in cells stimulated at 1 Hz increases in isolated cells from diabetic mice (Casis et al., 2000). These cells shown, phase 1 (early repolarization) less pronounced and a longer phase 2 (plateau). Otherwise, the resting membrane potential and the AP amplitude were unchanged (Casis et al., 2000). Moreover, T2DM models (*db/db* mice, a leptin receptor mutant that develops obesity, diabetes, hyperinsulinemia and increased levels of triglycerides and fatty acids; Buchanan et al., 2005) shown similar prolongation of action potentials (Figure 4) (Shimoni, 2001).

Prolongation of action potentials is due to, at less in part, by a decrease in the output repolarizing currents (Ito and IK) (Shimoni et al., 1999, 2004; Qin et al., 2001). The amplitude of the currents (Ito and IK) are reduced in myocytes isolated from T1DM murine models (Torres-Jacome et al., 2013). In addition, myocytes isolated from *db/db* mice (12th week of age), as a T2DM model, exhibit a sustained attenuation of the output currents compared to the *db/+* mouse (Shimoni, 2001; Shimoni et al., 2004). Therefore, despite the dramatically different levels of insulin circulating in the STZ diabetic rat and *db/db* mouse, the changes of action potential and ventricular output K^+^ currents are similar, suggesting that these effects could be generated by an impaired ligand-receptor coupling or absence of insulin signaling pathway (Shimoni et al., 2004).

Despite the total output currents of K^+^ are impaired (Figure 4), is necessary to determine which one is most relevant electrical change. In dogs treated with alloxan (a toxic glucose analog that destroys the β-pancreatic cells) to induce T1DM, after 8 weeks, were evaluated the different currents contribution to the AP through voltage-clamp experiments. In this case, no changes were found in neither amplitude nor voltage-dependent IKr and ICa activation currents. Conversely, the amplitude of the Ito and IKur currents significantly decreased by 69.3 and 54.6% in myocyte cells isolated from diabetic models, respectively. In an effort to clarify the effects of insulin, after a week from developed diabetes, dogs were treated with insulin (0.5–1.0 IU/kg day-1s.c), and an increase in current density was evident only in the Ito and IKur currents (Lengyel et al., 2007). This result is consistent with findings obtained from myocytes isolated from hearts of diabetic mice (STZ model) incubated with insulin, in which the amplitudes of these currents were restored (Magyar et al., 1992; Xu et al., 1996, 2002; Shimoni et al., 1998).

Several animal models have been used to characterize the effects of diabetes on the heart (Shimoni et al., 2004; Boudina and Abel, 2010) and include a variety of systemic effects (Bugger and Abel, 2009). Diminished glucose oxidation rates in cardiomyocytes occur as early as 48 h after the induction of diabetes by STZ treatment, which is reversed by insulin treatment (Chen et al., 1984). Insulin-stimulated cardiac glucose uptake and their utilization are also impaired in type 2 diabetic hearts (Mazumder et al., 2004). Furthermore, in hyperinsulinemic animal models of T2DM, such as the Zucker diabetic rat, the impaired insulin signal transduction in cardiac muscle (Kolter et al., 1997) is also associated with diminished glucose utilization and increased fatty acid utilization in the heart (Chatham, 2001). In all of these models it is difficult to separate the relative contribution of intrinsic defects in cardiomyocyte insulin signaling from the conditions generated by the altered systemic metabolism, such as hyperglycaemia and hyperlipidaemia.

The cardiac-specific insulin receptor knockout (CIRKO) mice model, development by *cre/loxP* recombination to specifically inactivate insulin signaling in cardiomyocytes *in vivo*, preserve insulin signaling in other cells such as endothelial cells, VSMC, and liver and skeletal muscle cells (Brüning et al., 1998; Abel et al., 1999) and let to investigate the specific role of impaired cardiac insulin signaling without the additional conditions associated to systemic metabolic perturbations (Belke et al., 2002). CIRKO mice develop contractile dysfunction but not heart failure, associated with decreased glucose and fatty acid oxidation (Belke et al., 2002). Also develop mitochondrial dysfunction, with an increased mitochondrial superoxide production, and display fatty acid-induced mitochondrial uncoupling (Boudina et al., 2009). Likewise, CIRKO mice represent a useful model to dissect the mechanisms and the effects of insulin in the heart, in the absence of systemic effects of insulin deficiency, or the additional conditions associated to systemic effects of insulin treatment as occur in many models of diabetes.

### Effects of diabetes on gene expression of Kv channels

Decreased of outward currents induced by diabetes conditions may be attributed to deficient protein expression (Howarth et al., 2009), changes in biophysical properties, or both (Lopez-Izquierdo et al., 2014). Until now, there is no difference in voltage dependence of inactivation, inactivation kinetics, or recovery from inactivation state. It is suggested that these effects are explained by an abnormal protein expression (Lopez-Izquierdo et al., 2014).

The study of the cardiac gene expression by RT-PCR considering the genes: *kcnd2* and *kcnd3* encoding the Ito α-subunit Kv4.2 and Kv4.3, respectively (Frank-Hansen et al., 2005); *kcne2* encoding to MiRP1, Ito and IKs β-subunit (Roepke et al., 2008), on tissue extracted from left ventricles of mice with STZ-induced diabetes showed significant reductions of ~80% in the expression of *kcnd2* and *kcne2*, whereas no significant differences were shown for *kcnd3* (Howarth et al., 2009). These results were replicated in CIRKO mice, where KChIP2, Kv4.2 accessory subunit had about 50% reduction. Therefore, Kv4.2 and its assembly with specific accessory subunits such KChIP2 and MiRP1, would be relevant in maintaining transient K^+^ current, mainly in its fast component (Itof) (Lopez-Izquierdo et al., 2014).

Studies in animal models of diabetes also reveal a reduction in the mRNA and protein levels of the components of Itof (Qin et al., 2001). Based on the analysis of protein levels of ion channels evaluated by western blot obtained for CIRKO mice left ventricle, show a reduction of ~50% in the abundance of Kv4.2 and Kv1.5 (Lopez-Izquierdo et al., 2014). In contrast, other studies using a T1DM rat or CIRKO mice model, report a reduction in Kv4.2 protein expression, but not for the Kv4.3 channel, were neither the messenger nor protein expression were down-regulated (Kääb et al., 1998; Nishiyama et al., 2001). KChIP decreased by 40% when compared to the control group (Lopez-Izquierdo et al., 2014). Furthermore, immunofluorescence analysis shows a reduction of immunoreactivity for Kv4.2, Kv4.3, and Kv1.5 protein, in isolated ventricular cardiomyocytes from T1DM models vs. healthy controls, and no changes of Kv2.1 (IKr determining protein) were found (Torres-Jacome et al., 2013). Moreover, in extracted tissue from ventricles of diabetic animals evaluated by immunohistochemistry, a reduction has been shown in the number of cardiomyocytes with immunopositive reactivity to Kv4.2 and MiRP1 (IKur β-subunit), consistent with the reduced expression genes that encode them (Howarth et al., 2009). Therefore, a reduction in Kv4.2, Kv1.5 messenger and protein provides an explanation for the reduced Ito and IKur currents, observed in impaired insulin signaling conditions, respectively. At the same time, interaction with accessory subunits, such as KChiP2 and MiRP-1, would be relevant to the maintenance of these currents (Grubb et al., 2012).

In addition, the results previously mentioned suggest a regulatory role of insulin in the expression of different potassium channels in diabetic cardiomyocytes. Incubation of isolated myocytes with insulin can recover the protein expression of Kv4.2 and Kv1.5 channels, and also restores the amplitude of potassium currents when tested after 3, 4, 5, and 6 h incubation (Shimoni et al., 1998). In the same way, the use of protein synthesis blockers, such as cycloheximide, blocks these currents' recovery (Rozanski et al., 1998). Furthermore, the effect of insulin depends on the integrity of the cytoskeleton, hence protein traffic inhibitors, as cytochalasin D, are able to attenuate this effect (Shimoni et al., 1999; Yang et al., 2002). Presumably, it is necessary that newly synthesized channels can effectively translocate to the cell membrane to maintain these specific repolarizing currents.

## Clinical implications

Insulin is a hormone with pleiotropic physiological functions, so it is normal to conclude that deterioration of insulin signaling also has pleiotropic dysfunctions. In this review, we discuss two main mechanism that govern the cardiovascular physiology, one mechanism in endothelial cell related with L-arginine transport and NO bioavailability, and a second main mechanism related with the regulation of Kv channels on cardiomyocytes.

The physiological role of NO is well known in human vasculature, so the largest contribution of this review is the establishment that insulin resistance is closely related with potential reduction of hCAT-1 expression and/or activity in endothelial cells. Reduction of L-arginine transport is a primarily cause of endothelial dysfunction and induces a reduction of NO synthesis due to restriction of substrate availability for eNOS. Cardiovascular pathologies associated to insulin resistance are well documented, and we propose that the significant reduction of L-arginine transport mediated by lower expression and/or alteration of plasma membrane localization of the transporter could be a link between the impairment of insulin signaling and vascular disease. Clinical evidence shows that the forearm L-arginine uptake is reduced in subjects with essential hypertension or with family history of hypertension. In peripheral blood mononuclear cells (PBMCs) isolated from the same patients, a reduction was seen of *V*_max_ of L-arginine transport and lower sensitivity to Ach-dependent vasodilatation, parameter that was reverted by co-infusion of L-arginine (Schlaich et al., 2004). Also a reduction in agonist-induced blood flow was observed in response to insulin in patients with T2DM, an alteration that is associated to lower capacity of transport of L-arginine and NO synthesize (Rajapakse et al., 2013). Additionally, in CHF patients it is determined that there is a reduced capacity for transport of L-arginine from plasma to the tissues with reduction of transcardiac fractional extraction of L-arginine and lower expression of hCAT-1 in myocardial tissue, without changes in hCAT-2B expression. Also the transcardiac L-citrulline flux, as a parameter of NO synthesis in heart, is decreased in CHF patients (Kaye et al., 2002). These data indicate the presence of an abnormal L-arginine transport in CHF, suggesting a shared mechanism between endothelial dysfunction and heart disease, related with hCAT-1 down-regulation and impairment of NO synthesis.

Altogether, this information demonstrates that there is a positive feedback mechanism between insulin secretion, insulin signaling, L-arginine/NO pathway, and cardiac function that is disrupted in pathophysiological conditions such as metabolic syndrome, insulin resistance and diabetes.

In regards to the physiological role of insulin in the regulation of Kv channels and their association with cardiac electrical function specifically, this relationship would be given by an altered intracellular signaling in a similar way to the pathological processes involved in diabetes mellitus (Casis and Echevarria, 2004). Diabetic patients show electrocardiographic changes associated with a high incidence of cardiac arrhythmias (Veglio et al., 1999, 2002a,b, 2004). Specifically, long QT syndromes (duration of ventricular depolarization and repolarization of the myocardium) have been reported as a result of an increase in the time of the ventricular action potential. This phenomenon has been linked to a three-fold increased risk of sudden cardiac death (Straus et al., 2006). Even more, studies have shown a significantly increase in relative death risk for subjects with QTc (QTc, heart rate-corrected QT interval) longer than 0.43–0.44 s (Brown et al., 2001). This relationship has been shown for all cause mortality, cardiac mortality, and sudden death (Festa et al., 2000).

Prevalence of QTc prolongation has been evaluated in different cohorts of diabetic subjects (Veglio et al., 1999, 2002a,b) and in the National Health and Nutrition Examination Survey III (NHANES III) population (Brown et al., 2001; Zhang et al., 2011). In T1DM patients, the prevalence of an abnormally prolonged, corrected QT was 16% in the whole population, 11% in males and 21% in females. QTc in insulin-dependent diabetic female patients is longer than in male patients, even in the absence of diabetic complications known to increase the risk of corrected QT prolongation (Veglio et al., 1999). While in T2DM patients, prevalence of increased QTc duration was 25.8%, with no sex differences, showing a considerably high prevalence of increased QTc and their association with Coronary Heart Disease (Veglio et al., 2002a,b). Finally, in the NHANES III survey, the prevalence of QTc prolongation in diabetic subjects was nearly twice that of non-diabetic subjects (Brown et al., 2001; Zhang et al., 2011).

Different studies explain the action potential prolongation as a response to a reduction of repolarizing K^+^ currents, in cardiomyocytes isolated from type 1 to 2 diabetic models (Magyar et al., 1992; Shimoni et al., 1994; Casis et al., 2000; Shimoni, 2001; Torres-Jacome et al., 2013). To examine the effects of impaired insulin signaling on these electrophysiological changes, recent studies have measured the electrocardiographic activity in anesthetized CIRKO mice, in order to assess the effect of the lack of cardiomyocyte specific insulin receptor (Lopez-Izquierdo et al., 2014). When was compared the electrocardiographic recordings from CIRKO mice vs. control mice, it has been demonstrated a significant prolongation of the QRS, QT, and QTc intervals (Lopez-Izquierdo et al., 2014).

**Figure 5:**
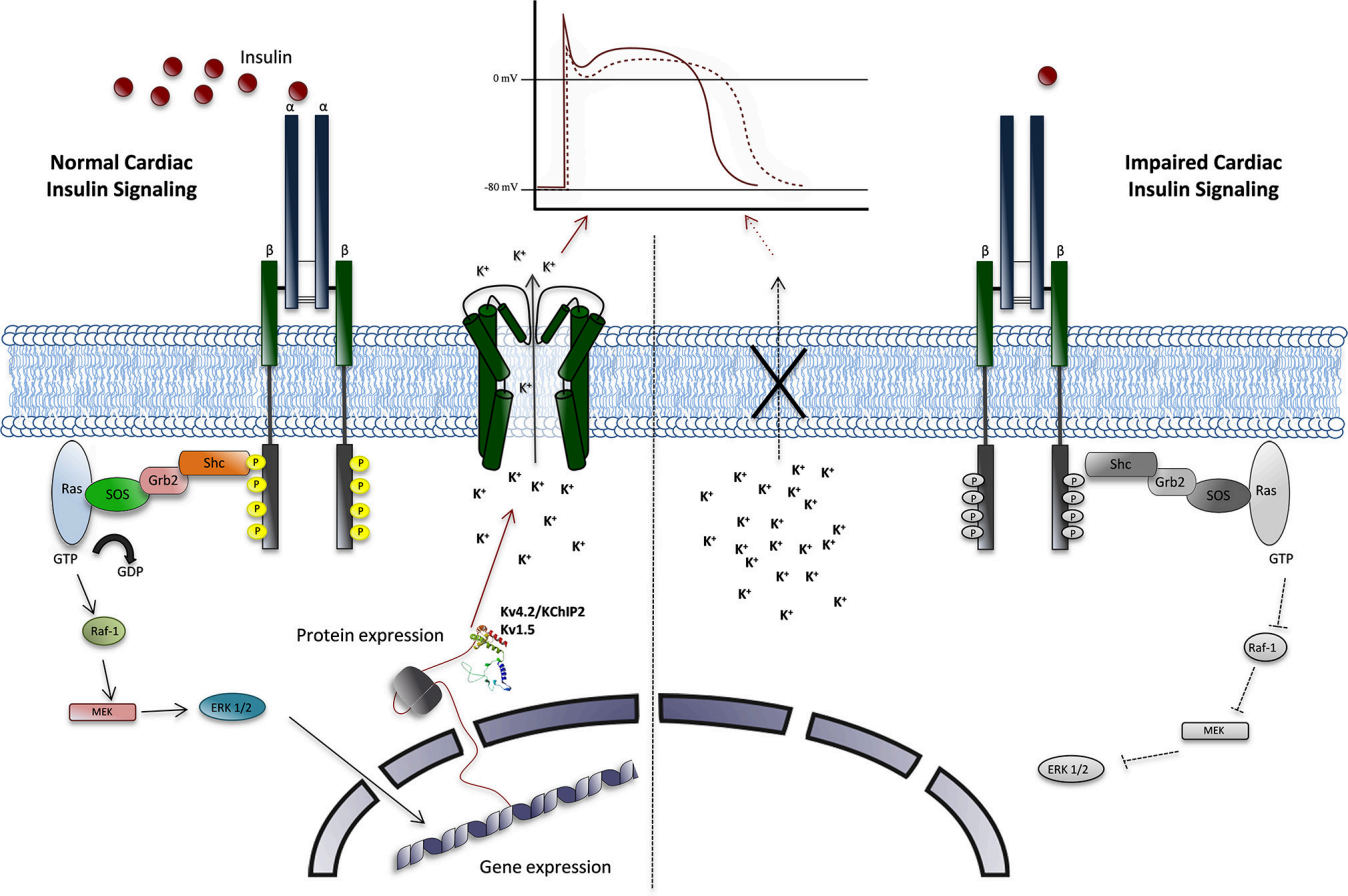
**Proposal model of Insulin regulation of cardiac Kv channels**. Normal cardiac Insulin signaling (right side) plays an important role in maintaining the repolarizing currents, in a Ras/MAP Kinase signaling pathway-mediated process. This regulation is achieved through gene expression and protein, which, specifically, determine the Kv4.2 and Kv1.5 α subunits, were need translocate to cellular membrane to generate Ito and IKur currents, which determine the appropriate action potential in ventricular cells. Furthermore, in disease, impaired insulin signaling (left side) and the reduce Ito and IKur currents amplitude, leads to an increase in the length of AP, manly in phase 2, due to the reduced availability of Kv channels with an increased risk of cardiac arrhythmias associated.

The specific contribution of impaired insulin signaling in gene expression for cardiac Kv channels, lead to a QT interval prolongation in experimental models (Figure 5) (Howarth et al., 2009). This is similar to the findings that were reported for diabetic patients (Veglio et al., 2002a,b). These results are independent of the systemic metabolic changes that join the disease.

## Concluding remarks

In summary, insulin has general effects on all tissues and a strong relationship with cardiovascular function. Specifically, through experimental models of impaired intracellular signaling, which occurs in pathological conditions such as diabetes, it is possible to elucidate the regulatory effects that tie this particular ligand to the vascular and cardiac function.

Diabetes involves a series of prevalent cardiovascular complications such as DCM, a disease that appears as relevant for studying the effects of insulin resistance. This is because it includes a number of pathophysiological events, which include the ventricular electric remodeling.

Insulin plays an important role in maintaining the current repolarizing, specifically the Ito and IKur components, which determine the appropriate action potential in ventricular cells. This regulation is achieved through genes and proteins expression, which determine the Kv4.2 and Kv1.5 α subunits, β subunits and KChIP2 and MiRP1 composition, in a Ras/MAP Kinase signaling pathway-mediated process (Figure 5).

Outside of metabolic function, it is also evidenced that insulin, through the lack of its signaling pathways, plays a regulatory role in the cardiac electrical activity through voltage-dependent potassium channels and can produce electrocardiographic changes, such as QT prolongation, and increased risk of sudden death in diabetic patients.

## Author contributions

SD and MG participated in bibliographic research, writing, edition, and figures design; DG and LC participated in bibliographic research in vascular effects; LS and LZ participate as vascular function and channels experts, respectively.

## Funding

This work was supported by Fondo Nacional de Desarrollo Científico y Tecnológico (FONDECYT 11100192, 1150377) and Vicerrectoría de Investigación y Desarrollo, Universidad de Concepción (VRID-Asociativo 213.A84.014-1.0), Chile.

### Conflict of interest statement

The authors declare that the research was conducted in the absence of any commercial or financial relationships that could be construed as a potential conflict of interest.

## Abbreviations

Ach: acetylcholine
AP: action potential
AsODNs: antisense oligonucleotides
BH_4_: tetrahydrobiopterin
Cav: voltage-gated calcium channel
CIRKO: cardiac-specific insulin receptor knock-out
DCM: Diabetic Cardiomyopathy
DM: Diabetes Mellitus
DOCK1: dedicator of cytokinesis protein 1
EC: electrocardiogram
eNOS: endothelial NO synthase
GAB1: Grb-2 associated protein
HAEC: human aortic endothelial cells
hCAT-1: cationic aminoacid transporter
HEKC: human embryonic kidney cells
HUVEC: human umbilical vein endothelial cells
IK: delayed rectifier potassium channel
IKr: rapidly activating delayed rectifier potassium channel
IKs: slowly activating delayed rectifier potassium channel
IKur: ultra-rapid potassium current
IR: Insulin receptor
IRS: insulin receptor substrate
Ito: transient outward potassium current
KChIP2: voltage-gated potassium (Kv) channel-interacting proteins
Kir: inwardly rectifying potassium channels
Kv: voltage-gated potassium channel
MAPK: Mitogen-activated protein kinases
MiRP: MinK-related peptide 1
Nav: voltage-gated sodium channel
NE: norepinephrine
NO: nitric oxide
PI3K: phosphatidylinositide-3-kinase
PKC: protein kinase C
QTc: heart rate-corrected QT interval
SHC: Src homology 2-domain containing protein
STZ: streptozotocin
T1DM: Type 1 Diabetes Mellitus
T2DM: Type 2 Diabetes Mellitus
Tyr: tyrosine amino acid
WHO: World Health Organization.
